# A role for immunohistochemical stains in perinatal brain autopsies

**DOI:** 10.1093/jnen/nlae019

**Published:** 2024-03-04

**Authors:** Angela N Viaene

**Affiliations:** Department of Pathology and Laboratory Medicine, The Children’s Hospital of Philadelphia, Philadelphia, Pennsylvania, USA; Department of Pathology and Laboratory Medicine, The University of Pennsylvania Perelman School of Medicine, Philadelphia, Pennsylvania, USA

**Keywords:** Brain, GFAP, Iba-1, Immunohistochemistry, Perinatal, White matter

## Abstract

Identification of central nervous system injury is a critical part of perinatal autopsies; however, injury is not always easily identifiable due to autolysis and immaturity of the developing brain. Here, the role of immunohistochemical stains in the identification of perinatal brain injury was investigated. Blinded semiquantitative scoring of injury was performed on sections of frontal lobe from 76 cases (51 liveborn and 25 stillborn) using H&E, GFAP, Iba-1, and β-APP stains. Digital image analysis was used to quantify GFAP and Iba-1 staining. Commonly observed pathologies included diffuse white matter gliosis (DWMG) and white matter necrosis (WMN). DWMG scores were very similar on H&E and GFAP stains for liveborn subjects. For stillborn subjects, DWMG scores were significantly higher on GFAP stain than H&E. β-APP was needed for identification of WMN in 71.4% of stillborn subjects compared to 15.4% of liveborn subjects. Diffuse staining for Iba-1 within cortex and white matter was positively correlated with subject age. Staining quantification on digital image analysis was highly correlated to semiquantitative scoring. Overall, GFAP and β-APP stains were most helpful in identifying white matter injury not seen on H&E in stillborn subjects. Immunostains may therefore be warranted as an integral part of stillborn brain autopsies.

## INTRODUCTION

Examination of the central nervous system (CNS) is an important component of a complete perinatal autopsy. In addition to evaluating brain maturation and development, CNS examination allows for the detection of injury to the developing brain. While hemorrhages, larger disruptions, and deformations may be readily apparent by gross examination, identification of more subtle, microscopic forms of CNS injury is equally crucial to a thorough and accurate postmortem examination. However, there are several factors that can hamper detection of perinatal CNS injury.

Several patterns of CNS injury are unique to the perinatal period (20 weeks gestation to 28 postnatal days), up through the first year of life. Understanding the different patterns of injury, which regions of the brain are most likely to be affected, and how to identify these pathologies on gross and microscopic examination contributes to the complexity of performing perinatal CNS autopsies. Both perinatal white matter injury and gray matter injury can be difficult to detect on gross examination, particularly in the acute stages, necessitating sampling and microscopic examination of regions where injury most frequently occurs.

The locations where neuronal injury occurs are unique in developing brains. Structures such as the basis pontis and subiculum may be affected (pontosubicular necrosis) with or without obvious neuronal injury in cortex (1, 2). Additionally, the histology of perinatal gray matter injury varies depending on developmental stage, with neuronal apoptosis predominating in the weeks leading up to term before transitioning to the hypereosinophilic neurons with pyknotic nuclei seen postnatally (3). Unique patterns of white matter injury also exist in the perinatal period. Examples of such white matter pathologies include white matter necrosis ([WMN], sometimes referred to as periventricular leukomalacia), and diffuse white matter gliosis ([DWMG], also known as telencephalic leukoencephalopathy and gliotic encephalopathy) (4).

Thorough gross and microscopic examination can be hampered in the perinatal period by the immaturity of the developing brain as well as autolysis, particularly in stillborns. Developing brains are fragile due to immaturity of vascular connective tissues and a relative dearth of long-range neuronal connections (5, 6). Additionally, immature brains contain several proteolytic enzymes that degrade CNS tissues after death (7–9). In instances of death *in utero*, the deceased fetus may remain at body temperature for a prolonged period of time, accelerating the effect of proteolysis. Eventually, CNS tissue becomes fragmented and even liquefied. Microscopically, autolysis can result in nuclear disintegration, cytoplasmic swelling, and architectural disruption, hindering evaluation of some pathologic processes.

Despite these challenges, identifying perinatal CNS injury is crucial for correlating with clinical history, radiology studies, and postmortem findings in other organs, including the placenta. Though not always possible to ascertain, the relative timing of injury prior to death can offer important clinical insights and may play a role in medicolegal cases centered around labor and delivery. Recent studies suggest that immunostains such as glial fibrillary acidic protein (GFAP) may be better at highlighting white matter injury than hematoxylin and eosin (H&E), although this was not systematically examined (10, 11). Therefore, a potential role for immunohistochemical stains as an integral part of perinatal CNS postmortem examination was investigated. Staining for GFAP (marker of astrocytes which can be used to identify DWMG), ionized calcium binding adaptor molecule1 ([Iba-1], marker of macrophages and microglia which proliferate in response to CNS injury), and β-amyloid precursor protein ([β-APP], marker of axonal injury), was assessed and compared to H&E examination, in order to identify instances in which immunohistochemical stains provide important diagnostic information beyond routine H&E examination alone.

## MATERIALS AND METHODS

The study was granted exemption by the Children’s Hospital of Philadelphia Institutional Review Board. Illuminate InSight software (Softek Illuminate, Inc., Overland Park, KS) was used to query an institutional database of pathology reports for autopsy cases in which the brain was examined. Liveborn and stillborn cases with microscopic examination of the brain were assessed to identify autopsies for which sections of frontal lobe containing cortex, superficial subcortical white matter, and periventricular white matter were taken. Cases with available formalin-fixed paraffin-embedded tissue blocks were selected for further study and included 51 liveborn and 25 stillborn brains. Clinical information was obtained from review of autopsy reports and the electronic medical record. For liveborn subjects born prematurely, all ages were adjusted/corrected for analysis.

### Histologic review and scoring

**Figure 1.**
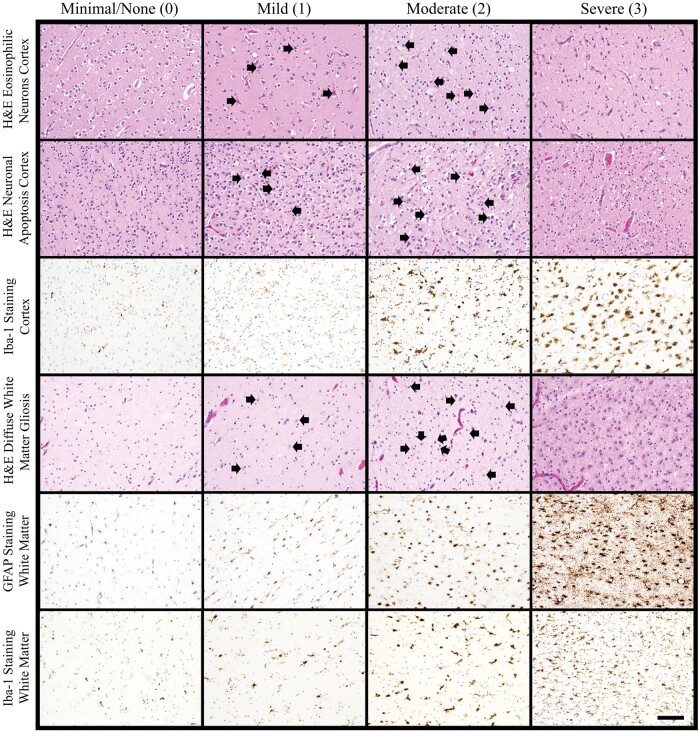
Histologic scoring for acute neuronal injury, diffuse white matter gliosis, GFAP staining, and diffuse Iba-1 staining. Example images are shown beneath the headers for the following scores: minimal/none [0], mild [1], moderate [2], and severe [3]. Acute neuronal injury within cortex included both neurons with eosinophilic cytoplasmic change and pyknotic nuclei (top row) and neuronal apoptosis (second row). Example images of acute neuronal injury are taken from sections of cortex stained with H&E at 200× magnification. No histologic signs of acute neuronal injury are seen in the minimal/none images while scattered neurons with eosinophilic cytoplasm and pyknotic nuclei (top row) or nuclear apoptosis (second row) are present for the mild and moderate images (black arrows). Nearly all neurons show evidence of acute neuronal injury in the examples of severe injury. Diffuse Iba-1 staining within cortex shows increasing numbers of microglia with increasing reactivity from left to right (third row, all images at 200× magnification). For diffuse white matter gliosis scoring on H&E (fourth row; all images taken at 200× magnification), reactive astrocytes are not apparent in the minimal/none image while scattered reactive astrocytes are seen in the mild and moderate images (black arrows). Numerous, widespread reactive astrocytes are present in the example image of severe injury. Immunohistochemical staining for GFAP to highlight reactive astrocytes is shown in the fifth row at 200× magnification; all images are taken within the frontal lobe subcortical white matter. Scoring was performed in a similar manner to diffuse white matter gliosis scores on H&E stain. Diffuse Iba-1 staining within the white matter (bottom row; all images at 200× magnification) was scored similarly to diffuse Iba-1 staining in cortex. The scalebar in the bottom right image corresponds 0.1 mm and applies to all images.

**Figure 2.**
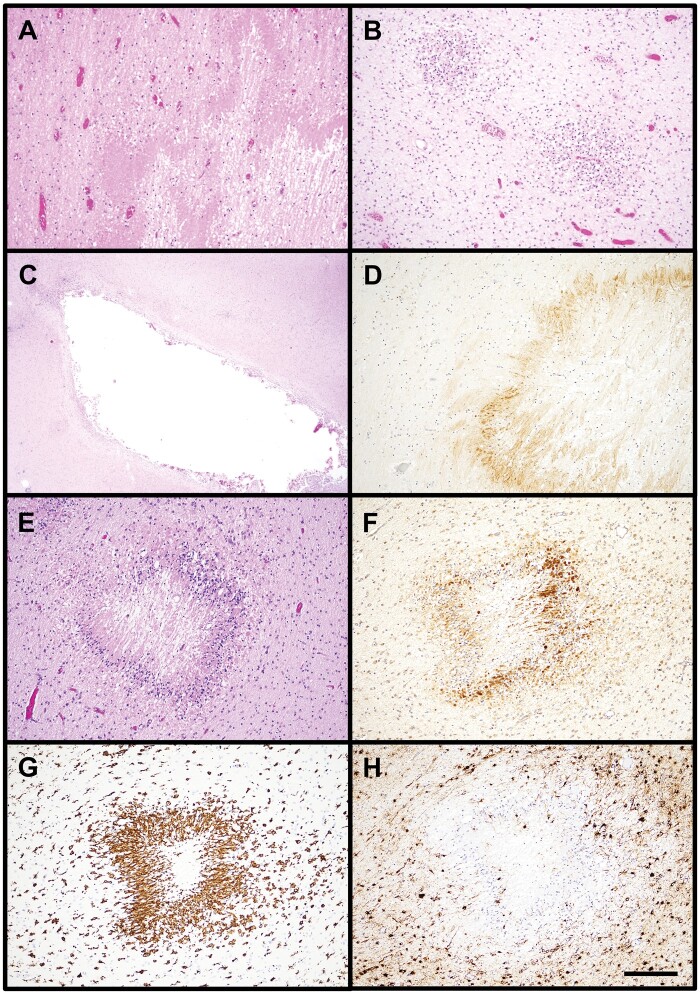
Examples of white matter necrosis. **(A)** A focus of acute white matter necrosis seen on H&E as a region of white matter with loss of nuclear detail (H&E stain, 100× magnification). **(B)** Two foci of subacute white matter necrosis characterized by collections of macrophages (H&E stain, 100× magnification). **(C)** Chronic, cystic white matter necrosis seen as a region of cavitation within the white matter. A few collections of macrophages are present along the periphery of the cavity (H&E stain, 20× magnification). **(D)** A focus of white matter necrosis highlighted by surrounding staining for β-APP (β-APP immunostain, 100× magnification). **(E)** A microscopic focus of white matter necrosis with surrounding axonal damage and gliosis seen on H&E (H&E stain, 100× magnification). **(F)** The same microscopic focus of white matter necrosis as shown in panel (E) labeled with β-APP staining (β-APP immunostain, 100× magnification). **(G)** The microscopic focus of white matter necrosis shown in panel E is also highlighted on Iba-1 staining (Iba-1 immunostain, 100× magnification). **(H)** GFAP immunostain labels reactive astrocytes surrounding the microscopic white matter necrosis shown in panel (E) (GFAP immunostain, 100× magnification). The scalebar in H applies to all images, corresponding to 0.2 mm for A, B, D, E, F, G, and H and 1.0 mm for C.

H&E-stained sections of frontal lobe underwent blinded review by a single reviewer with expertise in perinatal neuropathology (A.N.V.). Microscopic injury was scored on a semiquantitative scale as previously described (11). Scoring of acute neuronal injury within the cortex was performed as follows: 0 = no/minimal acute neuronal injury; 1 = mild, focal acute neuronal injury; 2 = moderate, focal and/or multifocal acute neuronal injury; 3 = widespread, severe acute neuronal injury (Fig. 1; Supplementary Data Fig. S1). Both neurons with eosinophilic cytoplasm and loss of nuclear detail as well as neuronal apoptosis were counted as acute neuronal injury. DWMG was scored as follows: 0 = no/minimal gliosis; 1 = mild, diffuse gliosis with scattered reactive astrocytes seen throughout the cortical white matter; 2 = moderate, diffuse gliosis with several reactive astrocytes seen throughout the cortical white matter; 3 = severe, diffuse gliosis with numerous reactive astrocytes seen throughout the cortical white matter (Fig. 1; Supplementary Data Fig. S1). Care was taken to evaluate only reactive astrocytes (eccentrically located nuclei and plumper, eosinophilic cytoplasm) and exclude myelination glia (smaller, hyperchromatic nuclei and elongated cytoplasm, Supplementary Data Fig. S1). WMN in any stage (acute, subacute, and chronic) was documented as being present or absent on H&E. Because necrosis varies histologically depending on survival time, the following histologic features were used to identify WMN: pyknosis and/or loss of nuclear staining for the majority of cells, collection(s) of macrophages, and cavitation (Fig. 2). The size of WMN was measured microscopically as the largest diameter of the necrotic focus on either H&E or β-APP stain (see below).

### Immunohistochemical staining and scoring

Immunohistochemical stains were also performed on the same sections of frontal lobe evaluated on H&E stain. Five-µm unstained sections were cut from formalin-fixed paraffin-embedded tissue blocks and stained with the antibodies to the following: GFAP (M0761, 1:600, Dako, Glostrup, Denmark), Iba-1 (019-19741, 1:2000, FUJIFILM-Wako, Richmond, VA), and β-APP (MAB348, 1:1000, Millipore, Burlington, MA). Blinded scoring of immunostains was then performed separately from H&E evaluation in order to avoid any potential influence on immunostain examination. Diffuse GFAP staining within the cortical white matter was scored on a semiquantitative scale analogous to scoring of DWMG on H&E stain as described above: 0 = none/minimal; 1 = mild; 2 = moderate; 3 = severe (Fig. 1). Focally increased collections of reactive astrocytes were excluded from DWMG scoring, and evaluation was focused on widespread/diffuse astrocyte reactivity throughout the white matter rather than focal gliosis, which may be encountered in the setting of WMN. Diffuse Iba-1 staining was scored separately for the cortex and white matter (again excluding localized foci of increased staining) as follows: 0 = none/minimal; 1 = mild; 2 = moderate; 3 = severe (Fig. 1). Staining for β-APP was scored as positive or negative for axonal injury, indicating a focus of WMN, regardless of the size or number of positive foci (Fig. 2D, F). Only focal/regional β-APP staining was counted; diffuse staining within the white matter was excluded in the absence of strong, focal staining.

### Digital image analysis

**Figure 3.**
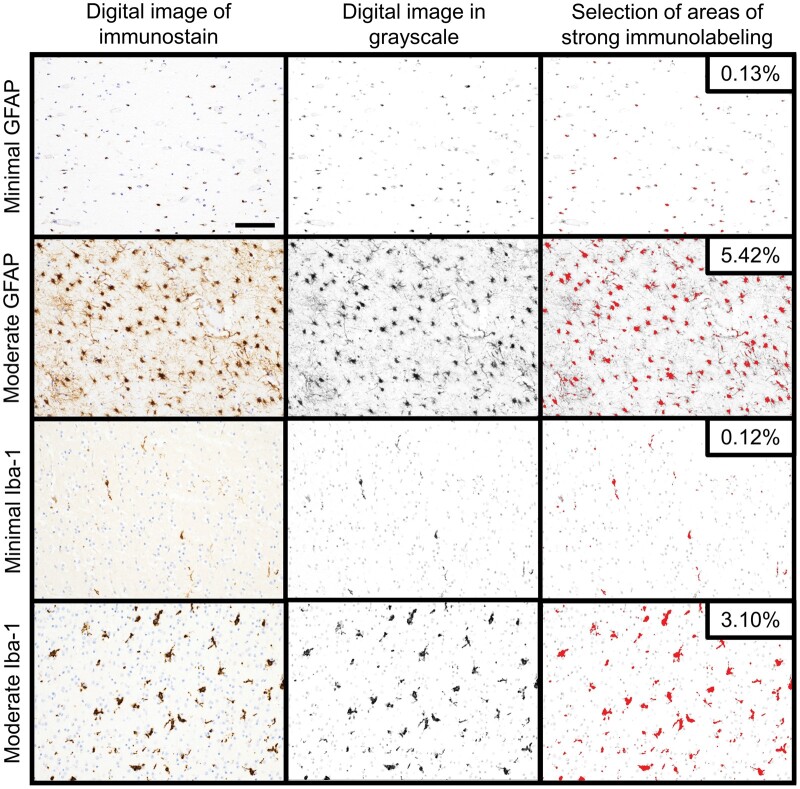
Digital image analysis of diffuse GFAP and Iba-1 staining. For each subject, digital images from regions of representative diffuse GFAP and Iba-1 staining were taken at 200× magnification (GFAP staining was evaluated in the white matter, and Iba-1 staining in cortex and white matter were evaluated separately). Representative examples of subjects with minimal and moderate GFAP staining in the white matter as well as subjects with minimal and moderate Iba-1 staining within cortex are shown in the left column. These images were converted to grayscale (middle column). The right column shows the result of blinded adjustment of thresholding performed to highlight regions with strong immunolabeling. The calculated percent area of the image with strong immunolabeling is shown for each of these example cases in the upper right-hand corner of the image. The scalebar in the upper left photomicrograph corresponds to 0.1 mm and applies to all images.

For each case, representative photomicrographs of GFAP and Iba-1 staining at 200× magnification were taken (DS-Fi3, Nikon, Tokyo, Japan) and analyzed using ImageJ software (National Institutes of Health, Bethesda, MD) (12). Images were converted to grayscale, and blinded adjustment of thresholding was performed to highlight regions with strong immunolabeling (Fig. 3). The percent area of the image with immunolabeling was then calculated. Measurements of percent area of labeling were performed separately for diffuse GFAP staining within the white matter, diffuse Iba-1 staining within the cortex, and diffuse Iba-1 staining within the white matter.

### Statistical methods

Statistical analysis was performed using SPSS version 28.0 (IBM Corp. Armonk, NY). Statistical significance was defined as p < .05 and based on 2-tailed t-tests, Mann-Whitney tests, and regression analyses. Standard deviation (SD) is reported as the mean ± SD.

## RESULTS

A total of 76 cases were selected for evaluation and staining (51 liveborn and 25 stillborn, Tables 1 and 2). The liveborn subjects had a mean adjusted age of 40 weeks gestation ± 12 weeks (range 22 weeks gestation–36 weeks postnatal); the stillborn subjects had a mean age of 36 weeks gestation±4 weeks (range 23–41 weeks gestation) (p < .05). The stillborn subjects had a significantly longer mean postmortem interval than did the liveborn subjects (81.9 ± 57.4 hours vs 47.0 ± 44.4 hours, respectively; p < .01). The male to female ratios for the liveborn and stillborn subjects were 1:1 and 1:1.1, respectively.

**Table 1.** Demographics and neuropathologic findings for liveborn subjects

						Cortical injury		Diffuse white matter injury			White matter necrosis			
Subject no.	GA at birth (weeks)	Lifespan	Sex	PMI (hours)	Autolysis	ANI score (H&E)	Diffuse Iba-1 Score	DWMG score (H&E)	DWMG score (GFAP)	Diffuse Iba-1 Score	WMN (H&E)	WMN (β-APP)	Microscopic/macroscopic	Size (mm)
**No/minimal white matter GFAP and Iba-1 reactivity**														
LB 1	24	1.5 weeks	F	40	None/mild	0	0	0	0	0	N	N	–	–
LB 2	25	3 days	F	48	None/mild	0	0	0	0	0	N	N	–	–
LB 3	26	1 day	F	44	None/mild	0	0	0	0	0	N	N	–	–
LB 4	30	8 weeks	M	33	Severe	3	2	1	0	0	N	N	–	–
**Diffuse white matter injury without WMN**														
LB 5	22	1 hour	M	40	None/mild	0	0	0	1	0	N	N	–	–
LB 6	22	3 weeks	M	36	None/mild	0	0	0	0	1	N	N	–	–
LB 7	25	2 months	F	120	None/mild	1	2	0	0	1	N	N	–	–
LB 8	25	7 months	M	42	None/mild	0	2	1	1	2	N	N	–	–
LB 9	26	2 days	F	36	None/mild	1	1	1	1	1	N	N	–	–
LB 10	26	3 days	F	44	None/mild	0	0	1	1	1	N	N	–	–
LB 11	26	5 months	M	10	None/mild	0	1	2	2	2	N	N	–	–
LB 12	27	6 months	M	72	None/mild	1	2	0	1	1	N	N	–	–
LB 13	28	1 week	M	65	None/mild	0	1	0	0	1	N	N	–	–
LB 14	28	3 weeks	M	110	None/mild	0	1	0	1	1	N	N	–	–
LB 15	29	8 months	F	10	None/mild	0	3	1	1	3	N	N	–	–
LB 16	30	3 months	M	70	Severe	0	3	3	3	2	N	N	–	–
LB 17	33	2.5 weeks	M	18	None/mild	0	0	0	0	1	N	N	–	–
LB 18	34	4 hours	F	43	None/mild	0	0	1	1	0	N	N	–	–
LB 19	34	5 days	M	48	None/mild	0	0	1	1	1	N	N	–	–
LB 20	35	7 weeks	F	3	None/mild	0	1	2	2	1	N	N	–	–
LB 21	35	2 months	F	24	None/mild	0	1	2	3	1	N	N	–	–
LB 22	36	1 week	F	41	None/mild	0	2	1	1	1	N	N	–	–
LB 23	36	2 months	M	120	None/mild	1	2	1	1	2	N	N	–	–
LB 24	37	10 minutes	F	72	None/mild	0	1	1	1	2	N	N	–	–
LB 25	37	1 day	M	204	None/mild	0	0	1	2	1	N	N	–	–
LB 26	37	2 days	M	21	None/mild	1	3	2	2	2	N	N	–	–
LB 27	37	3 days	M	5	None/mild	0	1	2	1	1	N	N	–	–
LB 28	37	1 week	F	16	None/mild	3	3	3	2	3	N	N	–	–
LB 29	37	6 weeks	M	41	None/mild	0	1	1	1	2	N	N	–	–
LB 30	37	7 weeks	M	44	None/mild	0	2	1	1	3	N	N	–	–
LB 31	37	3 months	M	12	None/mild	1	2	1	1	2	N	N	–	–
LB 32	37	3.5 months	M	24	None/mild	0	1	2	2	1	N	N	–	–
LB 33	38	2 months	M	58	None/mild	0	1	2	2	1	N	N	–	–
LB 34	38	4 months	F	34	None/mild	0	1	1	1	1	N	N	–	–
LB 35	40	1 hour	M	39	None/mild	1	1	1	2	1	N	N	–	–
LB 36	40	1 day	M	23	None/mild	0	1	2	2	1	N	N	–	–
LB 37	40	1 day	F	48	None/mild	0	1	1	2	2	N	N	–	–
LB 38	40	8 months	F	8	None/mild	1	2	0	0	2	N	N	–	–
**Diffuse white matter injury with WMN**														
LB 39	24	3 weeks	F	160	None/mild	0	0	0	1	0	Y	Y	Microscopic	1.3
LB 40	24	8 months	F	30	None/mild	0	2	1	1	2	Y	Y	Macroscopic	3.2
LB 41	26	9 months	F	26	None/mild	0	3	0	1	3	N	Y	Microscopic	0.6
LB 42	31	9 months	F	25	None/mild	3	2	3	2	3	Y	Y	Macroscopic	6.4
LB 43	35	1.5 weeks	F	28	None/mild	0	2	3	3	3	Y	Y	Microscopic	1.3
LB 44	36	1 week	F	28	None/mild	0	2	3	3	3	Y	Y	Microscopic	0.7
LB 45	36	1 week	F	3	None/mild	0	1	1	1	3	Y	Y	Microscopic	0.7
LB 46	36	1 week	M	80	None/mild	0	1	2	1	1	Y	Y	Microscopic	0.9
LB 47	37	30 minutes	M	7	None/mild	2	2	2	2	1	Y	Y	Macroscopic	1.2
LB 48	37	3 days	F	21	None/mild	2	3	3	3	2	Y	Y	Microscopic	0.6
LB 49	37	4 days	F	192	Severe	0	1	2	2	1	Y	Y	Microscopic	0.2
LB 50	37	3 months	M	24	None/mild	0	1	1	2	1	Y	Y	Macroscopic	1.5
LB 51	40	4 months	M	9	None/mild	3	3	1	0	3	N	Y	Microscopic	0.6

Subjects are grouped by pattern of white matter injury: no/minimal GFAP and Iba-1 reactivity, diffuse white matter injury without WMN, and diffuse white matter injury with WMN.

ANI, acute neuronal injury; DWMG, diffuse white matter gliosis; F, female; GA, gestational age; GFAP, glial fibrillary acidic protein; H&E, hematoxylin and eosin stain; Iba-1, ionized calcium binding adaptor molecule1; LB, liveborn subject; M, male; N, no (no white matter necrosis); PMI, postmortem interval; WMN, white matter necrosis; Y, yes (white matter necrosis present); β-APP, β-amyloid precursor protein; –, not applicable.

**Table 2.** Demographics and neuropathologic findings for stillborn subjects

					Cortical injury		Diffuse white matter injury			White matter necrosis			
**Subject no.**	**GA at birth (weeks)**	**Sex**	**PMI (hours)**	**Autolysis**	**ANI score (H&E)**	**Diffuse Iba-1 Score**	**DWMG score (H&E)**	**DWMG score (GFAP)**	**Diffuse Iba-1 Score**	**WMN (H&E)**	**WMN (β-APP)**	**Microscopic/macroscopic**	**Size (mm)**
**No/minimal white matter GFAP and Iba-1 reactivity**													
SB 1	32	M	108	None/mild	0	0	0	0	0	N	N	–	–
SB 2	34	M	35	None/mild	0	0	0	0	0	N	N	–	–
**Diffuse white matter injury without WMN**													
SB 3	23	F	12	Severe	0	0	0	1	1	N	N	–	–
SB 4	26	M	68	Severe	1	1	0	2	0	N	N	–	–
SB 5	33	F	70	None/mild	0	2	0	2	1	N	N	–	–
SB 6	34	F	28	Severe	0	1	1	2	1	N	N	–	–
SB 7	36	F	159	Severe	0	0	0	2	0	N	N	–	–
SB 8	36	M	240	Severe	0	0	0	1	0	N	N	–	–
SB 9	37	M	89	None/mild	1	2	1	2	2	N	N	–	–
SB 10	37	F	20	None/mild	0	0	0	2	2	N	N	–	–
SB 11	37	M	120	None/mild	0	0	1	3	1	N	N	–	–
SB 12	38	M	90	None/mild	1	0	1	2	2	N	N	–	–
SB 13	38	F	90	None/mild	0	1	0	2	1	N	N	–	–
SB 14	38	M	60	Severe	1	1	1	3	1	N	N	–	–
SB 15	39	M	168	None/mild	1	1	1	1	1	N	N	–	–
SB 16	39	F	192	Severe	0	0	0	3	0	N	N	–	–
SB 17	40	F	24	None/mild	0	1	1	2	2	N	N	–	–
SB 18	41	F	95	None/mild	1	1	1	2	1	N	N	–	–
**Diffuse white matter injury with WMN**													
SB 19	33	M	92	Severe	0	2	0	2	1	N	Y	Microscopic	0.5
SB 20	35	F	24	None/mild	0	0	0	2	1	Y	Y	Microscopic	0.6
SB 21	36	M	72	Severe	2	1	1	3	2	N	Y	Microscopic	2.7
SB 22	37	F	48	None/mild	0	0	1	3	1	N	Y	Microscopic	1.5
SB 23	40	M	48	None/mild	0	1	2	2	1	N	Y	Microscopic	0.5
SB 24	40	F	50	None/mild	0	2	1	3	3	Y	Y	Macroscopic	1.0
SB 25	40	F	47	None/mild	0	1	2	3	1	N	Y	Microscopic	0.9

Subjects are grouped by pattern of white matter injury: no/minimal GFAP and Iba-1 reactivity, diffuse white matter injury without WMN, and diffuse white matter injury with WMN.

ANI, acute neuronal injury; DWMG, diffuse white matter gliosis; F, female; GA, gestational age; GFAP, glial fibrillary acidic protein; H&E, hematoxylin and eosin stain; Iba-1 = ionized calcium binding adaptor molecule1; M, male; N, no (no white matter necrosis); PMI, postmortem interval; SB, stillborn subject; WMN, white matter necrosis; Y, yes (white matter necrosis present); β-APP  =  β-amyloid precursor protein; –, not applicable.

### DWMG: H&E and GFAP staining

**Figure 4.**
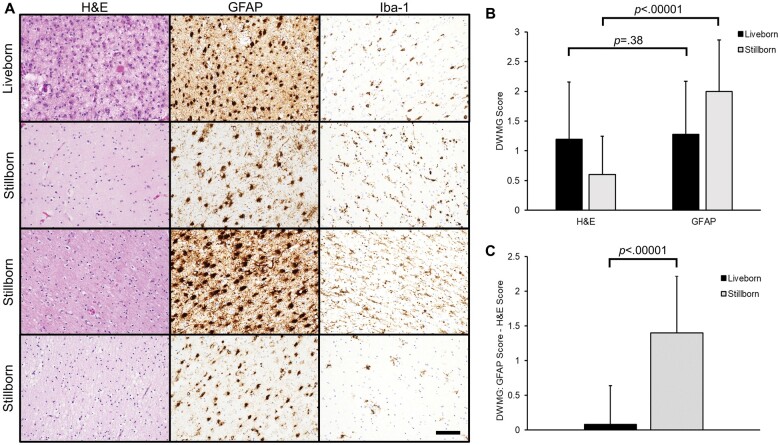
Diffuse white matter gliosis (DWMG) semiquantitative scores. **(A)** Examples of 1 liveborn and 3 stillborn subjects with moderate to severe DWMG. For the liveborn subject (top row), severe DWMG is seen on both H&E and GFAP stains. Iba-1 staining is also shown. The bottom 3 rows contain examples of the stillborn subjects for whom the DWMG is not readily apparent on H&E stains but is notable on GFAP stains. Iba-1 staining highlights reactive microglia which do not demonstrate the abundant cytoplasm observed in the GFAP-reactive astrocytes. All images are taken from the same region of the white matter for each subject at 200× magnification. The scalebar in the bottom right image corresponds to 0.1 mm and applies to all photomicrographs. **(B)** Bar graph showing the means of semiquantitative scoring for DWMG on H&E and GFAP stains for the liveborn (black bars) and stillborn (gray bars) subjects. **(C)** Bar graph depicting the mean difference in DWMG severity on GFAP and H&E stains (DWMG: GFAP score—H&E score) for the liveborn (black bars) and stillborn (gray bars) subjects. For panels (B) and (C), statistical significance is shown above the graphs, and error bars represent 1 SD.

Severity of DWMG was scored on both H&E and GFAP stains (Fig. 4). For liveborn subjects, the DWMG score was similar on H&E (1.19 ± 0.96) and GFAP (1.27 ± 0.90) (Z = −0.88, p = .38) (Fig. 4A, B). However, for stillborn subjects, DWMG scores were significantly higher on GFAP (2.00 ± 0.87) than H&E (0.60 ± 0.65) (Z = −4.02, p < .00001) (Fig. 4A, B). The score for DWMG on H&E was subtracted from the GFAP DWMG score for each subject to measure the difference in DWMG severity between the 2 stains (DWMG GFAP score—DWMG H&E score). Stillborn subjects showed significantly larger staining differences than did liveborn subjects (1.40 ± 0.81 vs 0.07 ± 0.56, Z = −5.38, p < .00001) (Fig. 4C).

Possible explanations for the differences observed in DWMG scores between H&E and GFAP stains for the stillborn subjects were investigated. The difference in DWMG scoring (DWMG GFAP score—DWMG H&E score) was compared to subject age and postmortem interval. No statistically significant correlations between the difference in DWMG score and subject age (r = 0.18; p = .13) and postmortem interval (r = 0.22; p = .06) were observed. Severe autolysis was more frequently observed in stillborn (36.0%) than liveborn (5.9%) subjects, and stillborn subjects with severe autolysis had slightly higher average differences in DWMG (GFAP-H&E) scores than those with no/minimal autolysis (1.78 ± 0.67 vs 1.19 ± 0.83, respectively) though this was not statistically significant (Z = −1.44, p = .15). The possibility that GFAP staining could be seen in cells other than reactive astrocytes (e.g. within the cytoplasm of macrophages) was considered. Iba-1 stains were reviewed for cases in which there were discrepancies between DWMG scores on GFAP and H&E. In all cases, Iba-1 staining did not highlight macrophages or microglia with morphology that would suggest concurrent GFAP staining in these cells (i.e. the Iba-1-positive cells appear to have smaller amounts of cytoplasm and different morphologies than the GFAP-positive cells; Fig. 4A).

### Diffuse Iba-1 staining

Scoring of diffuse Iba-1 staining was positively correlated with other histologic markers of injury. Within the cortex, the amount of Iba-1 staining increased with severity of acute neuronal injury observed on H&E stains (r = 0.25, p < .05). Similarly, within the white matter, diffuse Iba-1 staining was positively correlated with the amount of DWMG seen on both H&E (r = 0.39, p < .001) and GFAP (r = 0.46, p < .001) stains.

Overall, more diffuse Iba-1 staining was seen in liveborn than stillborn subjects. This finding was best observed within cortex (liveborn subjects: 1.33 ± 0.96, stillborn subjects: 0.72 ± 0.73; Z = −2.47, p < .05) with a similar trend present in the white matter (liveborn subjects: 1.45 ± 0.94, stillborn subjects: 1.04 ± 0.79; Z = −1.64, p = .10). The amount of diffuse Iba-1 staining increased with subject ages, showing a significant positive correlation in both the cortex (r = 0.58, p < .001) and white matter (r = 0.53, p < .001). Diffuse Iba-1 staining was not significantly correlated with postmortem interval (r = 0.17, p = .14).

### WMN and β-APP

**Figure 5.**
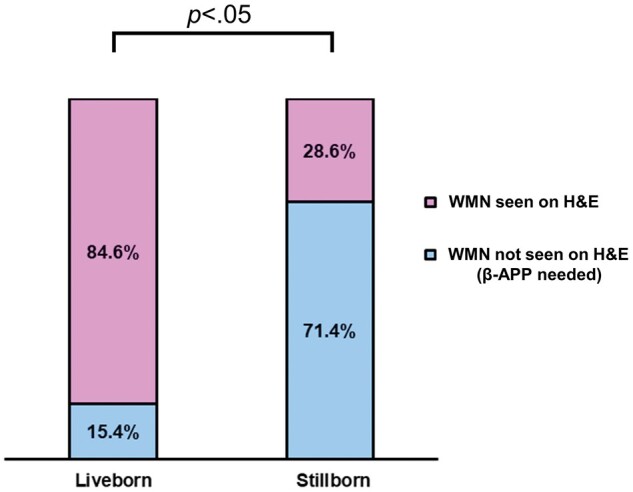
White matter necrosis (WMN) detection on H&E and β-APP stains. For liveborn and stillborn subjects with WMN, the percentage of subjects for which WMN was apparent on H&E is shown in pink, and blue represents the percentage of subjects for which β-APP staining was needed to identify the WMN. Percentages for each group are shown within the bars. Statistical significance is shown above the graph.

The presence or absence of WMN was evaluated on H&E and β-APP stains. WMN was present in 25.5% of liveborn cases and 28.0% of stillborn cases (χ^2^ = 0.11, p = .74). However, for cases in which WMN was present, it was seen at higher rates on H&E in liveborn (84.6%) than stillborn (28.6%) subjects. Conversely, when looking at all cases with WMN, β-APP was needed for identification of this lesion in 71.4% of stillborn subjects compared to 15.4% of liveborn subjects (χ^2^ = 4.10, p < .05) (Fig. 5). Moderate to severe DWMG was seen in 100% of stillborn subjects with WMN and 53.8% of liveborn subjects with WMN.

### Acute neuronal injury

Acute neuronal injury within cortex was observed in 27.5% of liveborn subjects and 28.0% of stillborn subjects and was often mild (average scores were 0.47 ± 0.90 and 0.32 ± 0.56 for liveborn and stillborn subjects, respectively). Moderate to severe acute neuronal injury was present in 7 cases (6 liveborn and 1 stillborn subject) and was seen in association with WMN in 5 cases (71.4%). Mild acute neuronal injury was noted in 14 cases with DWMG in the absence of WMN.

### Digital image analysis of GFAP and Iba-1 stains

**Figure 6.**
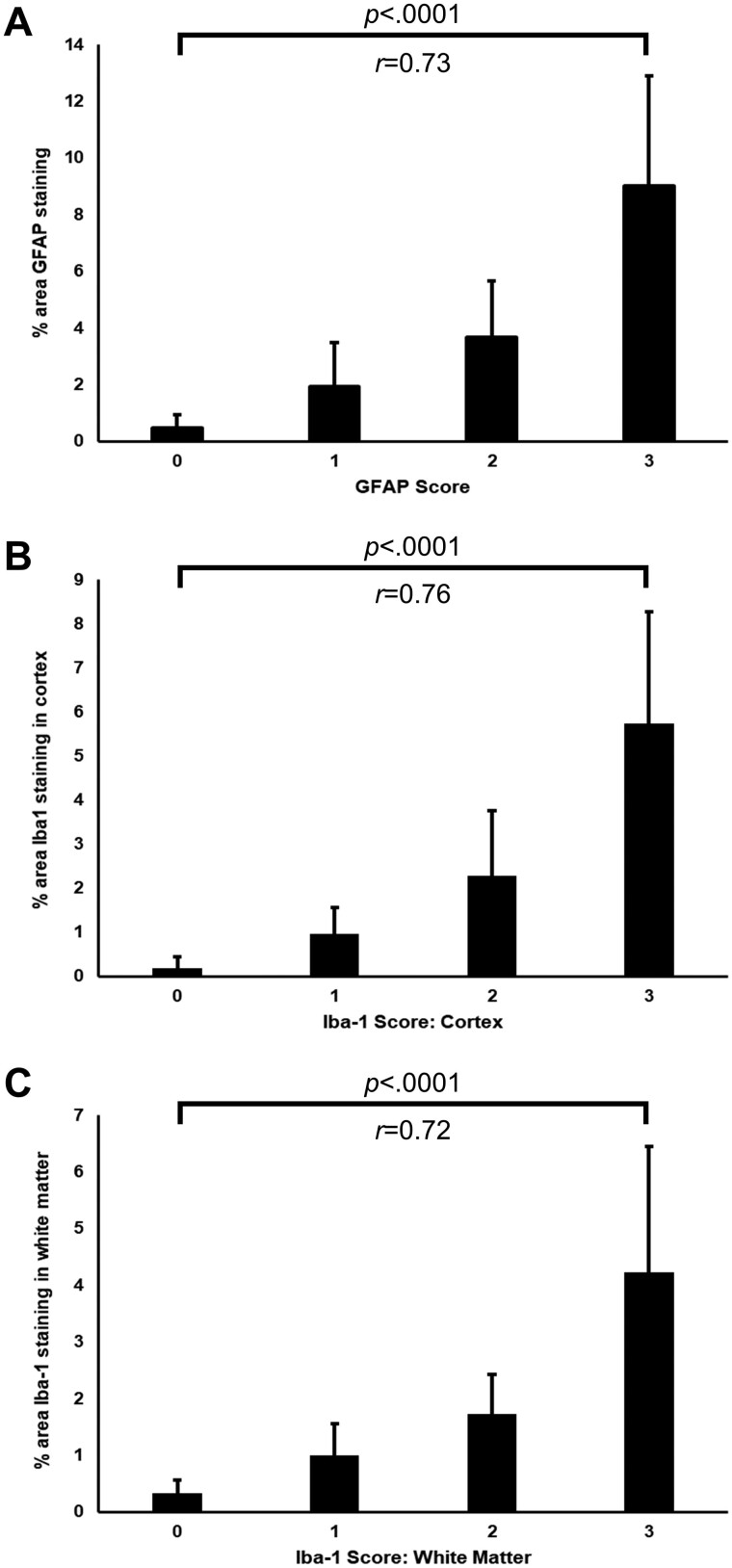
Comparison of semiquantitative scores and percent area immunolabeling. **(A)** Bar graph showing the relationship between semiquantitative GFAP scoring for diffuse white matter gliosis and digital image analysis of percent area staining for GFAP within the white matter. The mean percent area of GFAP immunolabeling is shown for each semiquantitative score (0–3). **(B)** Bar graph showing the relationship between semiquantitative Iba-1 scoring within the cortex and digital image analysis of percent area staining for Iba-1 within cortex. The mean percent area of Iba-1 immunolabeling is shown for each semiquantitative score (0–3). **(C)** Bar graph showing the relationship between semiquantitative Iba-1 scoring within the white matter and digital image analysis of percent area staining for Iba-1 within the white matter. The mean percent area of Iba-1 immunolabeling is shown for each semiquantitative score (0–3). Error bars represent SD. Statistical significance and correlation coefficients are shown above each graph.

The percent area of staining for GFAP and Iba-1 was evaluated using digital image analysis. Semiquantitative scoring of GFAP was strongly correlated to the percent area of GFAP staining by digital image analysis (r = 0.73, p < .0001) (Fig. 6A). Similar findings were observed for Iba-1 staining. Strong positive correlations between semiquantitative scoring of diffuse Iba-1 staining and percent area of Iba-1 labeling were seen in cortex (r = 0.76, p < .0001) and white matter (r = 0.72, p < .0001) (Fig. 6B, C). Mirroring what was observed on semiquantitative Iba-1 scoring, there was higher percent area of Iba-1 labeling in liveborn subjects than stillborn subjects in both the cortex (liveborn: 1.73%±1.90%, stillborn: 0.50%±0.67%; p < .001) and the white matter (liveborn: 1.68%±1.70%, stillborn: 0.89%±0.68%; p < .05).

## DISCUSSION

A small panel of immunostains was investigated to determine whether the stains highlighted perinatal CNS injury that was not detectable on routine H&E evaluation. Overall, GFAP and β-APP were found to be the most helpful in identifying white matter injury that was not readily apparent on H&E in stillborn subjects. Scores for DWMG were significantly higher on GFAP than on H&E for stillborn subjects whereas gliosis scores were very similar between these 2 stains in liveborn subjects. β-APP was necessary to detect acute WMN in a significant subset of stillborn subjects. While a small portion of liveborn subjects also required β-APP staining for diagnosis of WMN, this histologic finding was apparent on H&E in the majority of cases.

Age was considered a potential factor in the discrepancy seen in DWMG on H&E and GFAP stains. However, no significant correlation between subject age and difference in DWMG score on GFAP and H&E was seen. One major difference between the stillborn and liveborn subjects was postmortem intervals, which were significantly longer in the stillborns. Stillborn subjects tended to exhibit more severe autolysis than liveborn subjects (36.0% vs 5.9% of cases, respectively), which can hamper H&E evaluation. Though there was a slight positive correlation between postmortem interval and the differences in DWMG staining between GFAP and H&E, it was not statistically significant (p = .06). One possible explanation is that postmortem interval is only one factor that contributes to the severity of autolysis. The amount of time between death *in utero* and delivery as well as the temperature at which a decedent is maintained prior to autopsy both contribute to the autolytic process. Severe autolysis and tissue fragmentation are diagnostic challenges that may make identification of reactive astrocytes or early WMN difficult to identify. These data confirm that both microglial activation and gliosis can be identified in stillborn cases with severe autolysis (Table 2). On average, stillborn subjects with more severe autolysis had larger differences in DWMG scores on GFAP than H&E stains than did subjects with no/minimal autolysis, though this difference was not statistically significant (p = .15). An example of a stillborn case with severe autolysis is shown in the bottom row of Figure 4A; here, GFAP was helpful in highlighting reactive astrocytes that were difficult to detect in the autolyzed tissue on H&E stain.

Although age was not significantly correlated to discrepancies in H&E and GFAP scoring of DWMG, it is worth noting that the immaturity of developing brains can present a challenge to identifying DWMG. Astrocytes capable of producing a hypertrophic response are detectable in fetal brains at around 20 weeks gestation (13). However, reactive astrocytes in the perinatal period are not as pronounced as what is observed later in life, i.e. they are smaller and without the same abundance of eosinophilic cytoplasm that is observed in older children and adults. Myelination glia are also present at certain timepoints in the extended perinatal period and can sometimes be difficult to distinguish from astrocytes on H&E. Myelination glia have smaller nuclei with more elongated cytoplasm than reactive astrocytes (Supplementary Data Fig. S1B), and express markers of immature oligodendroglial lineage such as O4 without appreciable GFAP labeling (14). Additionally, the pattern of GFAP staining can be helpful as it highlights the star-shaped morphology of reactive astrocytes, which is not observed in developing and mature oligodendrocytes (15). However, it is important to note that GFAP is expressed in precursor cells that give rise to oligodendrocytes (15, 16). The possibility that GFAP labeling could be seen in microglia/macrophages was considered, particularly in cases where DWMG was apparent on GFAP but not H&E. However, in these cases, the morphology of cells observed on GFAP stains was not present on Iba-1 stains, supporting the interpretation that the GFAP labeling was indeed predominantly within reactive astrocytes.

White matter injury, including WMN, can be detected on β-APP staining within hours after an insult (17), and has been reported to be detectible within 30 minutes in the setting of severe head injury (18, 19). β-APP staining is useful in the early detection of WMN and may be observed prior to histologic signs of necrosis on H&E. Iba-1 can also label WMN (Fig. 2G) if adequate time for microglial activation has passed (20, 21). In this study, when WMN was present, it was detected on H&E in the majority of liveborn subjects compared to a minority of stillborn subjects. While autolysis hindering H&E evaluation may factor into this discrepancy, the timing of CNS injury prior to death could also play a role. In the liveborn subjects, there may have been longer survival intervals between the insult(s) that resulted in white matter injury and death for the WMN to become apparent on H&E; however, due to the complex medical histories of many of these subjects, definitive timing of the critical injury cannot be determined in most cases. Indeed, not only acute, but subacute and chronic WMN were all observed in liveborn subjects, whereas WMN in stillborn subjects tended to be acute. For many stillborn subjects, the duration of survival between the insult and demise may not have been sufficient for acute WMN to become apparent on H&E, necessitating the use of β-APP for diagnosis.

WMN was present in 71.4% of cases with moderate to severe acute neuronal injury, mirroring prior work that reported a strong correlation between neuronal loss and WMN (14). In the 2 cases of severe neuronal injury without WMN, (i.e. WMN was not observed on gross examination or microscopically), there was moderate to severe DWMG. Mild acute neuronal injury was also observed in the setting of DWMG without WMN. Diffuse white matter injury, in the form of DWMG and/or diffuse microglial activation, was seen in all cases of WMN.

The amount of diffuse staining for Iba-1 was positively correlated with the scoring of histologic signs of CNS injury. This was true for H&E stains in both the cortex (acute neuronal injury) and white matter (DWMG) and for GFAP staining within the white matter (DWMG), consistent with microglial activation in the setting of brain injury. However, the amount of Iba-1 staining was also found to increase with age over the examined extended perinatal period in both cortex and white matter. A similar pattern of staining for microglia has been previously described (22). Increased Iba-1 labeling over the extended perinatal period confounds the interpretation of diffuse Iba-1 staining. However, Iba-1 and related immunohistochemical stains that can be used to identify microglia/macrophages (e.g. CD68, CD163, and HLA-DR), can still be very useful in highlighting focal injury in the CNS including WMN.

GFAP and Iba-1 stains were interpreted and scored on a semiquantitative scale corresponding to no staining, mild staining, moderate staining, and severe/extensive staining. As a means to validate this semiquantitative scoring, a quantitative assessment of percent area of positive immunolabeling was also performed using digital image analysis. Since β-APP staining was evaluated as present/absent, corresponding digital image analysis was not performed for that stain. Semiquantitative scoring for both diffuse GFAP and Iba-1 staining was highly correlated to the quantitative digital image analysis results. This finding supports pathologists’ ability to reliably interpret the extent of both diffuse GFAP and Iba-1 immunolabeling in perinatal brain autopsies.

Several studies have shown that that not only cystic WMN, but also noncystic WMN and DWMG contribute to long-term neuropsychological disabilities (23–33). Although the greatest risk period for perinatal white matter injury is 24–32 weeks gestational age, the same patterns of injury can be observed in late-preterm and even term infants, particularly in those with congenital heart disease or other risk factors for intrauterine insults (34–36). In the United States, 9.8% of infants are born preterm (prior to 37 weeks gestational age), and the survival rate is 81.4% for infants born prior to 32 weeks gestational age (37, 38). Given the high rates of adverse neurodevelopmental outcomes in individuals with perinatal white matter injury (39), understanding the underlying pathophysiology as well as identifying potential risk factors is of considerable importance. Additionally, with advances in the care of preterm infants, the patterns of CNS injury currently observed may differ from what was historically reported (40). Along with intrapartum and postnatal risk factors such as congenital heart diseases, lung diseases, and placental abruption (41–44), damage to the developing brain may also occur *in utero* (45–48). Given the findings presented in the current study, it is possible that there are additional risk factors and/or associations between *in utero* pathologies and white matter injury that could be elucidated with the use of select immunostains to highlight DWMG and acute WMN in stillborn subjects.

Resources for detailed perinatal autopsies are not always readily available and routine use of immunostains in all cases may not be practical. This work sought to identify stains that would be most useful and in which instances they should be considered. The results of this study indicated that GFAP and β-APP immunostains provided information beyond what could be obtained from H&E stains, particularly in stillborn subjects. In order to be most effective, immunostaining would ideally be performed in brain regions in which white matter pathologies are most likely to be observed. As DWMG is a diffuse process, this finding should be detectable on GFAP staining in any section containing an adequate sampling of the cerebral white matter. WMN most often occurs in the periventricular white matter, although it can be seen elsewhere within the cerebral white matter. The most common locations are anterior to the frontal horns, adjacent to the lateral corners of the lateral ventricles, and within the temporal acoustic and optic radiations (3, 49). For the purposes of the present study, sections of frontal cortex were used as they were the most routinely sampled regions that included cortex, superficial subcortical white matter, and periventricular white matter within a single tissue block. However, the other brain regions described above where WMN and DWMG can be observed should provide similar diagnostic information.

Immunostains are widely known to play an important part in supplementing H&E diagnosis in both surgical specimens and autopsy cases. The results of this study indicate that immunostaining can also play a useful role in perinatal CNS examination, particularly in stillbirths. GFAP stained DWMG that was often less pronounced or not detectable on H&E, and β-APP highlighted acute WMN not yet apparent on H&E. White matter injury was more easily detected on H&E stain in liveborn subjects though β-APP did show acute WMN not seen on H&E in a small number of subjects. While diffuse Iba-1 staining correlated with other histologic indicators of CNS injury, its interpretation is confounded by increased staining within the cortex and white matter over the developmental period examined. Therefore, in stillborn cases for which detection of perinatal CNS injury is critical, immunostaining for GFAP and β-APP in a strategically sampled region of cerebral white matter may be warranted for most accurate neuropathologic workup.

## Supplementary Material

nlae019_Supplementary_Data
