# Supplementary figures and images for: A role for immunohistochemical stains in perinatal brain autopsies
Source: J Neuropathol Exp Neurol. 2024 Mar 4;83(5):345–56. doi: 10.1093/jnen/nlae019 (PMC11029462; doi:10.1093/jnen/nlae019)

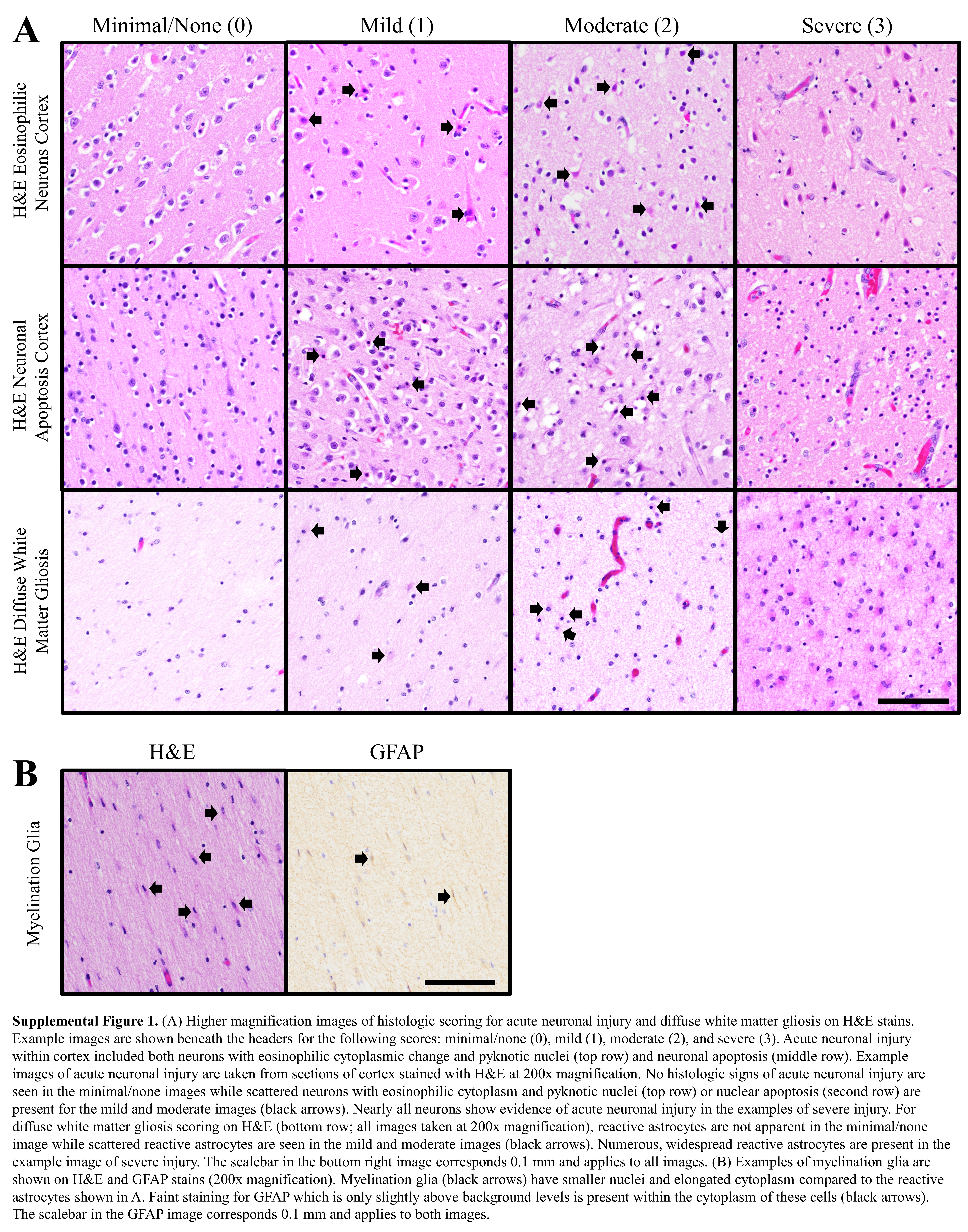

Supplement: nlae019_Supplementary_Data [file nlae019_supplementary_data.zip › nlae019_Supplementary_Data/Supplemental Figure 1.jpg]
